# Algorithms Based on CWT and Classifiers to Control Cardiac Alterations and Stress Using an ECG and a SCR

**DOI:** 10.3390/s130506141

**Published:** 2013-05-10

**Authors:** María Viqueira Villarejo, Begoña García Zapirain, Amaia Méndez Zorrilla

**Affiliations:** DeustoTech-Life Unit, DeustoTech Institute of Technology, University of Deusto, 48007 Bilbao, Spain; E-Mails: mviqueira@deusto.es (M.V.V.); mbgarciazapi@deusto.es (B.G.Z.)

**Keywords:** ECG, arrhythmia, extrasystole, heart rate, SCR, CWT, stress, cardiac alterations, signal processing, J48

## Abstract

This paper presents the results of using a commercial pulsimeter as an electrocardiogram (ECG) for wireless detection of cardiac alterations and stress levels for home control. For these purposes, signal processing techniques (Continuous Wavelet Transform (CWT) and J48) have been used, respectively. The designed algorithm analyses the ECG signal and is able to detect the heart rate (99.42%), arrhythmia (93.48%) and extrasystoles (99.29%). The detection of stress level is complemented with Skin Conductance Response (SCR), whose success is 94.02%. The heart rate variability does not show added value to the stress detection in this case. With this pulsimeter, it is possible to prevent and detect anomalies for a non-intrusive way associated to a telemedicine system. It is also possible to use it during physical activity due to the fact the CWT minimizes the motion artifacts.

## 1. Introduction

In recent years, there has been an increase in the number of remote health monitoring applications due to the importance of detecting anomalies and illness or diseases [1]. These devices acquire the signals permanently, not only at the specialist's office, and they can detect anomalies which may not appear in an ordinary medical revision.

Remote monitoring systems are not only used to detect anomalies, but they are also used by chronic patients to control their constants while at home, without the need to be in hospital, thus improving the patients' quality of life [2,3]. In this case, the system could act by warning the specialist about any problem detected [4]. In [5] Kurl *et al.* show that the duration of the QRS complex is related to cardiac death, which indicates that it is necessary to record the electrocardiogram (ECG) regularly to avoid sudden cardiac death episodes.

Within the field of monitoring, it is also important to detect the stress levels of a person because they can negatively affect health [6]. The described study uses a commercial ECG with the three following final objectives:

- Remote monitoring: acquisition of different signals for their posterior treatment.
- Detection of anomalies.
- Detecting whether a person is stressed or not.

For the third purpose, the different tests have been complemented with the Skin Conductance Response (SCR) in order to verify and compare the results. This study does not pretend to develop a system which differentiates objectively whether a person is stressed, but the intention is rather to find out whether is possible to control stress wearing only one commercial pulsimeter. The users' opinion has been taken into account in order to distinguish between stress and relax periods.

The acquired device communicates via Bluetooth because this is the most widely used technology in commercial devices. However, there are healthcare applications that communicate via ZigBee, as seen in [7,8]. The communication between the developed SCR and the control centre is via ZigBee.

As previously mentioned, one of the purposes of the final application is the detection of possible anomalies. For this study, the detection of extrasystoles and arrhythmias was chosen due to the fact that two of the participants presented these anomalies. In this case, the signals to analyse have been given by the ECG and the method used to analyse them is the Continuous Wavelet Transform (CWT) [9].

Nowadays, stress is one of the most common diseases due to current lifestyles: it is the body's reaction to pressure situations. Excessive stress can increase the risk of contracting diseases [10], so it is important to control some emotional states to reduce stress levels. Through an application which detects whether a person is stressed in real-time, it is possible to help that person to control this emotion by either changing the user's environment or teaching different methods on how to reduce stress. In this case the method used to detect stress levels is a non-invasive system which analyses different physiological signals measured by the SCR and the pulsimeter (specifically, the heart rate). To detect the emotional state of the user, it is necessary to perform previous trials to classify the different answers. The trials selected are relatively short because the final application should establish a stress threshold in a short period of time. Two different tests have been done, which are better described in the Materials and Methods section:

- Listening to annoying sounds: the user hears different sounds, which are supposed to be irritating. Each sound is separated by 15 seconds.
- Stroop test: this test is based on the difficulty of identifying the color described by a word when it is written in a different color. This test has been used in different studies to induce stress in people [11,12].

This study contains the following parts: first of all, the state of the art with different techniques and devices. Secondly it presents the different methods followed by the design and the results. Finally, it indicates the discussion and the different conclusions reached.

## 2. State of the Art

There exist numerous monitoring systems in order to record and analyse different biosignals remotely due to the advantages that they represent. In [13] the author explains different contents which are related to healthcare computing, including examples and the state of the art of different means of communication and the different devices used.

In [14] a solution which integrates different devices via Bluetooth is described, providing a solution for the monitoring of patients who suffer Chronic Heart Failure (CHF). Another solution for recording different signals is to integrate different sensors in one device, which would make it more comfortable for the user. The prototype in [15] integrates ECG, skin temperature, accelerometer and an optional photo-plethismograph (PPG). Gil *et al.*[16] show a developed system that measures EEG, ECG, respiration and PPG via Bluetooth.

Remote monitoring is also implemented in clothes, known as ‘smart textiles’. In [17] a shirt which combines an ECG and an accelerometer is presented. The accelerometer notices if the user has made a movement that can interfere in the ECG measurements. Smart clothes are not only used for monitoring different parameters, they are also used to interact with the user depending on the acquired signals. This way, it is possible to use them with autistic children or elderly people who are not able to express their emotions [18]. There are other wearable systems which acquire more data such as [19]: they use one shirt which measures ECG, PPG, BP, temperature, GSR and HR.

As said before, this study includes a stage which detects possible anomalies in the patient's ECG. In [20], Pandey and Mishra describe a method for detecting cardiac diseases from ECG. In the present study, the authors use QRS complex as a reference to analyse the ECG. There are numerous studies that indicate different methods and algorithms to detect it. In [21], Koheler *et al.* reviewed and compared different algorithms to detect the QRS complex. The ECG of the users can be affected by motion because the device will record the heart activity during the whole day. In [22], Benitez *et al.* describe an algorithm based on the Hilbert transform which differentiates the R wave, the T peak and the P wave with an average of 99.87% sensitivity while minimising the motion artefacts. The study described in [23] detects the QRS complex using the Support Vector Machine to remove baseline wander with 99.68% success.

In [24], the authors implement a method based on a Wavelet Transform to analyse TWA in order to predict the vulnerability of ventricular arrhythmia. Alvarado *et al.*[25] propose a method based on the CWT with splines taking various scales into consideration to detect the QRS complex, obtaining an accuracy of 99.5%.

Gautam and Kaur [26] use the wavelet transform to detect possible arrhythmias. They indicate T-wave end point identification is not necessary to evaluate T-wave abnormalities.

The Continuous Wavelet Transform has been used in other studies which analyse physiological signals. In [27], the authors present a method based on CWT to identify EEG seizure activities in order to detect epileptic events. In the study described in [28], Minchev *et al.* use CWT to analyse the EEG of different players, showing that the frequency bands change whether the user wins or loses. The study described in [29], analyses blood flow oscillations (BFO) to study their contribution to the skin vasodilatory response.

This study combines the monitoring and analysis of ECG and GSR. In [30] Brawner and Goldberg describe an experiment whose objective is to provide teachers with the physiological answers (GSR and ECG) of their students in order to find out the reaction to different questions.

The literature shows different methods and devices used in order to detect a person's stress levels. Initially, determining people's stress level was based in subjective questionnaires in which the person answered by giving their reactions to different situations. Nowadays by means of different sensors, it is possible to obtain objective answers in order to determine whether a person is stressed or not.

In [31] Healey and Picard describe methods of detecting stress levels in drivers with an accuracy of 90% by recording ECG, electromyogram (EMG), skin response and breathing rate (BR). In [32], Carter *et al.* study the relationship between muscle sympathetic nerve activity (MSNA), BP and HR. They also examine whether there are any differences between men and women. The heart rate variability (HRV) is acquired in [33] to detect stress parameters. The data were acquired from military personnel and the stressor factor was the training with the 9D5 Multi-place Underwater Egress Trainer. Jongyoon and Gutierrez-Osuna [34] propose removing the respiratory influences in HRV in order to obtain a better analysis.

In [35], Santos *et al.* work with Fuzzy Logic to analyse the response of different physiological signals using hyperventilation and talk preparation to induce stress. In [36] a study where the authors use the Support Vector Machine (SVM) to build a personalized stress detector subjecting the users to different tests is described. A real time system that monitors stress is presented in [37], using the Dynamic Bayesian Network (DBN) to model the stress of the user.

## 3. Materials and Methods

### 3.1. Pulsimeter

The BioHarness pulsimeter by Zephyr (city, country) was chosen because of the quantity of parameters the device is able to collect and because they provide the API for development. This pulsimeter has been used in other studies before [38]. The data needed by the application are within the following three frames:

- Breathing Waveform Packet: shows the variation of the user's respiration. Each sample is separated in time by 56 ms. The frame is sent every 1.008 seconds.
- ECG Waveform Packet: one of the functions of the pulsimeter is that of acting as a holter. This frame gives the data of the electrocardiogram. Each sample is separated by 4 ms and each frame is sent every 252 ms.
- Summary Data Packet: transmits numerous data. In this case, it provides information about skin temperature.

The resolution of the ECG is 0.013405 mV and the range is between 0 and 0.05 V.

### 3.2. SCR

**Figure 1.**
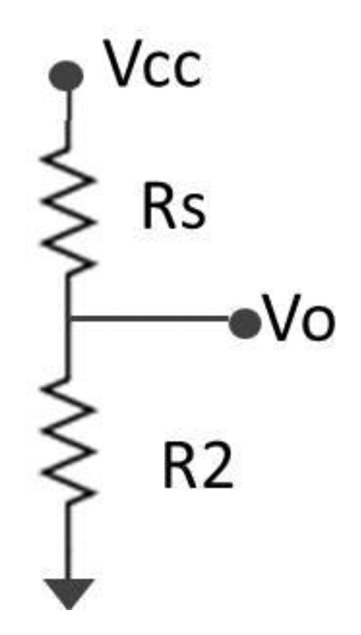
Voltage divider.

We have chosen to develop a device which is able to detect whether the skin resistance has varied. It does not directly measure the skin resistance, but another resistance is placed in series in order to measure the voltage. When a person is stressed, he sweats more and so there is an increment in the resistance of the skin. The different components of the device can be seen in [39]. Its A/D converter has a resolution of 0.573 mV and the maximum input voltage is 2.35 V. After comparing the real values measured after the voltage divider and the theoretic value, the error of each measurement is around 3.98%. The device uses a voltage divider, as it can be seen in Figure 1, to obtain the input tension Vo, which, after a low pass filter, is the input of the ADC.

In this case, Rs is the skin resistance and R2 is a resistance of 890 kΩ. The output voltage is obtained by:

(1) $$\text{Vo}=\frac{\text{R}2}{\text{Rs}+\text{R}2}\text{Vcc}$$

The value of Vo is inversely proportional to the value of Rs, thus, the output voltage when the user is stressed is higher than if he is not. The range of the skin response is between 10 kΩ and 10 MΩ and the measured voltages will be between 0.136 V and 1.755 V (9.93 MΩ). The sample frequency is 4 Hz. There is no need to know the exact value of the users' resistance since the present study only compares the output voltage when the subjects are stressed or relaxed. The obtained values are different depending on the user, so there is not a threshold that sets the limit between stressed or relax for the whole people. For each user, the application takes samples when the person is relaxed and when the person is submitted to stress, as well as makes a comparison between these two signals.

### 3.3. Matlab

Matlab was used for the programming of the initial user interface and for the subsequent analysis of the signals.

### 3.4. Eclipse

This integrated development environment was used to connect the control centre with different devices. The algorithms of communication were developed in C/C++.

### 3.5. Continuous Wavelet Transform (CWT)

This was used in order to analyse the ECG. The formula of the CWT is:

(2) $$C\left(a,b\right)=\frac{1}{\sqrt{a}}\int_{-\infty }^{\infty }x\left(t\right)\psi *\left(\frac{t-b}{a}\right)dt$$

*a* → scale
b → shift
*ψ* → mother wavelet
x (t) → original signal

### 3.6. Weka

This program was used in order to classify the samples of the SCR in the stress stage. The classifier used is J48.

### 3.7. Trials

Twelve users (seven men and five women) took part in this study. All the users were healthy, right handed and they work in the same department as the authors. User 2 presented arrhythmias in her ECG and user 5 presented extrasystoles. The characteristics of each user can be seen in Table 1.

The different users were asked to clean their hands before doing the test and, after waiting for five minutes to adapt the electrodes to the skin, the different tests started. For both tests, the users sat in front of a computer, with the SCR placed in the left hand. The tests took place in their work place, separated from the rest of the workers and with natural light, trying to adequate the test to the daily situation of the users. The electrodes used are Kendall/Tyco Arbo with a diameter of 24 mm.

**Figure 2.**
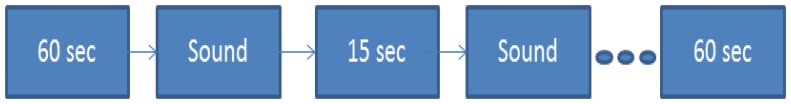
Sounds sequence.

**Figure 3.**
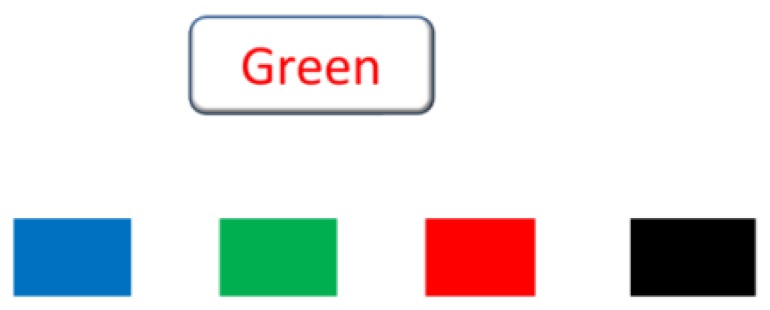
Stroop test 1.

**Figure 4.**
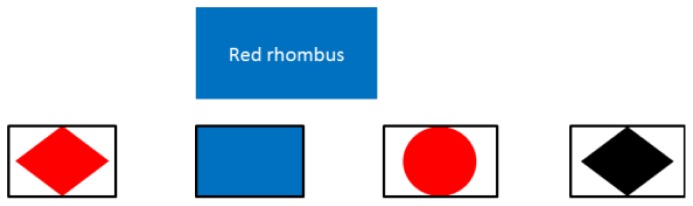
Stroop test 2.

**Figure 5.**
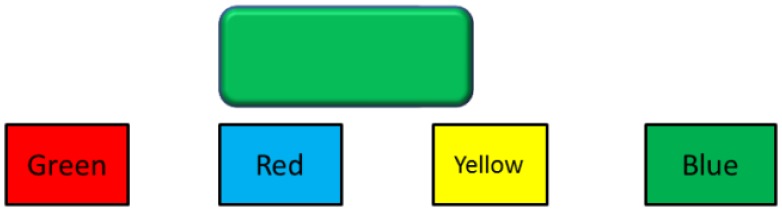
Stroop test 3.

Two different tests were carried out:

(1) Annoying sounds: as it can be seen in Figure 2, the user does not listen to any sounds during 60 seconds: after that, he listens to different sounds separated by 15 seconds, without listening to anything else again. At the end, the user stays another 60 seconds without listening to anything.
(2) Stroop test: the users have to select the correct option. There are three kinds of slides:
  - Stroop test 1: the user has to select the color that appears written. In Figure 3, the correct answer would be the green square
  - Stroop test 2: the solution is a combination of colors and figures. In the case of Figure 4, the correct answer would be the first figure (red rhombus).
  - Stroop test 3: the user has to choose the text that describes the color of the main rectangle. For Figure 5, the answer would be the first option (green)

**Figure 6.**
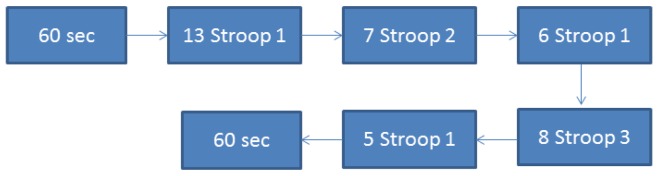
Stroop sequence.

The sequence of the slides is described in Figure 6:

Five seconds before the test starts, the screen shows a countdown in order to verify that the user is paying attention.

## 4. System Design

**Figure 7.**
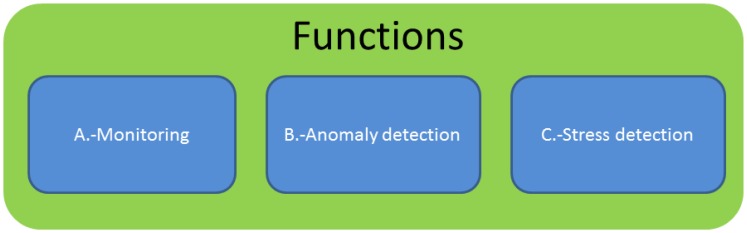
General block diagram.

Figure 7 shows the three differentiated blocks into which the study is divided: monitoring, anomaly detection and stress detection

### 4.1. Monitoring

**Figure 8.**
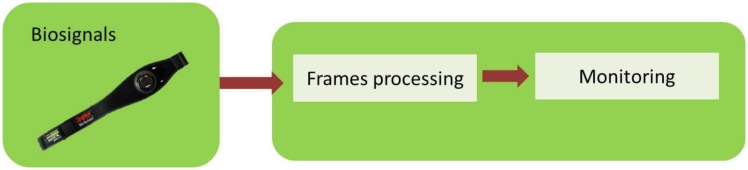
Block A—Monitoring.

A far as monitoring is concerned, the application collects the different frames from the pulsimeter for the posterior monitoring in the interface, Figure 8. This part does not include any subsequent processing of the signal.

**Figure 9.**
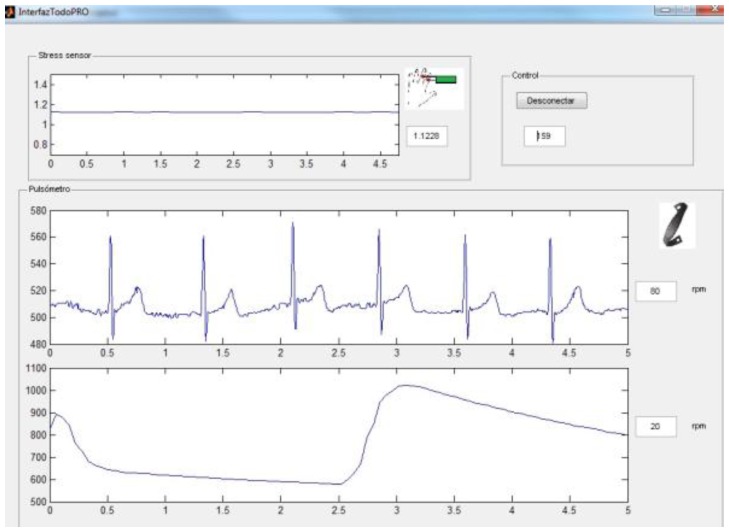
User's interface.

The appearance of the user interface can be seen in Figure 9 and it contains the SCR signals, the ECG, the heart rate, skin temperature and breathing wave.

**Figure 10.**
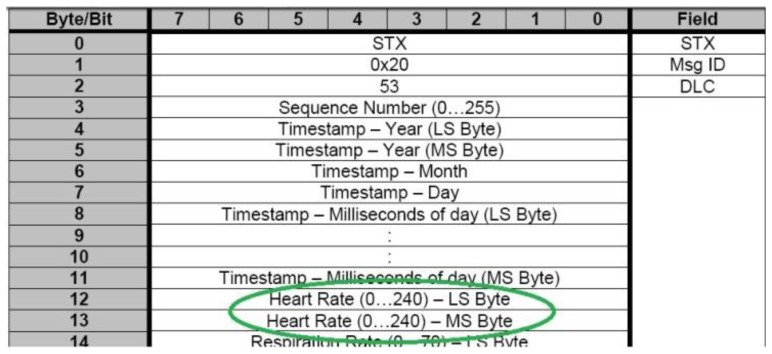
General Data Packet.

One of the most interesting parameters of monitoring is the heart rate of the user. The Zephyr pulsimeter gives this parameter in one frame. Figure 10 shows that bytes 12 and 13 contain the heart rate value.

### 4.2. Anomaly Detection

**Figure 11.**
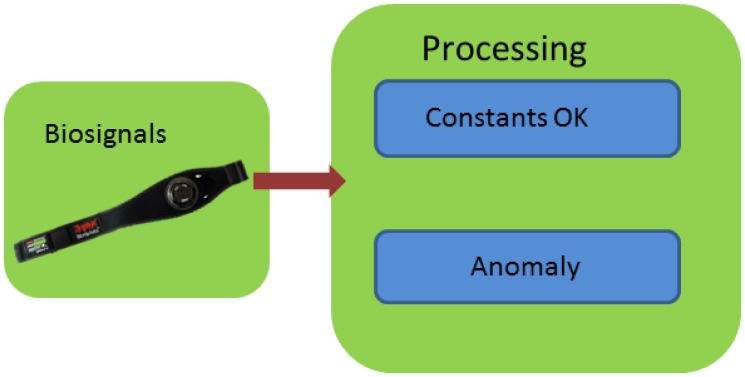
Block B—Anomaly detection.

This part is responsible for the detection of possible anomalies in the ECG; Figure 11 shows the general diagram:

As mentioned in the previous subsection, the heart rate monitoring is obtained by the General Data Packet. Instead of using this information, in this second block, we chose to extract the heart rate from the ECG since it is possible to analyse anomalies and the heart rate with the same frame.

**Figure 12.**
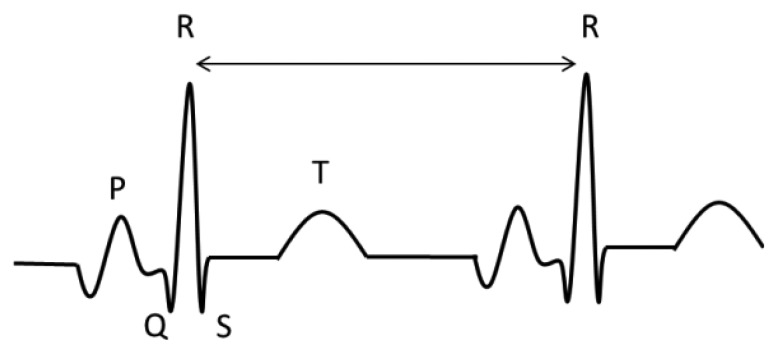
ECG components.

To analyse the ECG, we used the Continuous Wavelet Transform in order to find the R wave. Once this peak is detected it is possible to obtain the QRST features, according to the time intervals in each wave [40]. Figure 12 shows the principal waves of an ECG. To find the R peak, we used windows of five seconds.

**Figure 13.**
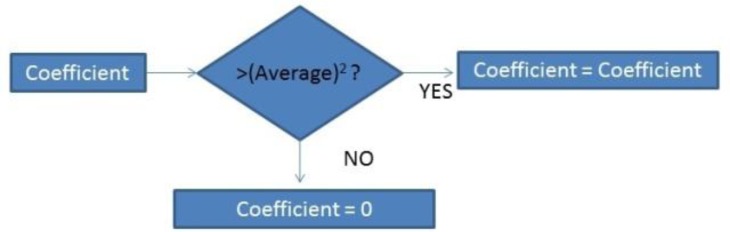
Algorithm.

Once the CWT is obtained, the coefficients are modified as Figure 13 indicates:

The chosen wavelet is coef5 as it is the one which produced the best results. esAfter that, the signal is normalized and the algorithm looks for different peaks. If the peak is superior to 60% of the average, it is taken as an R wave.

As one of the users (User 5) presents extrasystoles when she is relaxed, the algorithm was developed according to this anomaly. After doing some trials, it was established that there is one extrasystole if the peak is between 30% and 60%.

There can be cases in which the peak of the extrasystole is higher than 60%, so there is another stage which separates an R peak from an extrasystole peak. This stage executes the following algorithm:

if[t(i)−t(i−1)]⋅2.2<[t(i+1)−t(i)]→extrasystole

**Figure 14.**
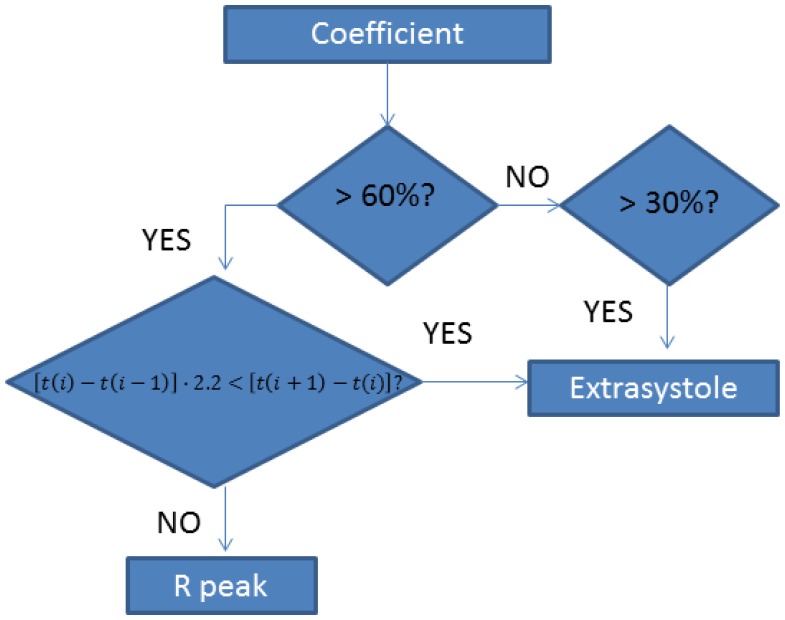
Algorithm for extrasystoles detection.

Figure 14 shows the different actions of the application to detect extrasystoles.

User 2 presented arrhythmia, so this anomaly has also been considered. In order to detect them it has been established that if the RR interval is 30% over or less than the interval before, there is an arrhythmia:

t(i)−t(i−1)≥1.3⋅[t(i−1)−t(i−2)]

or:

t(i)−t(i−1)⋅1.3≤[t(i−1)−t(i−2)]

**Figure 15.**
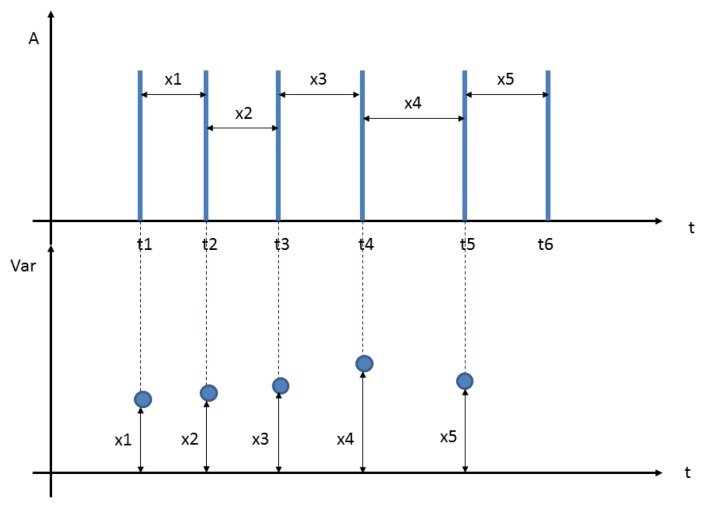
Variability method.

**Figure 16.**
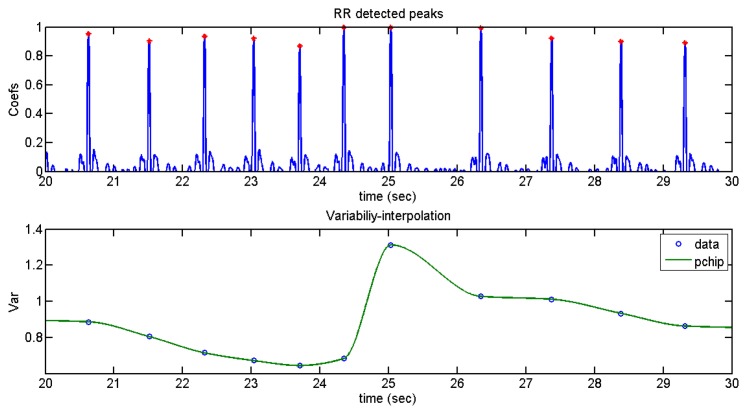
User 2 heart rate variability.

For this purpose, an analysis of the variability, as shown in Figure 15, was performed:

Subsequently, an interpolation of variability through cubic Hermite interpolation was carried out as can be seen in Figure 16.

It can be seen that there is arrhythmia between second 25 and second 26.

### 4.3. Stress Detection

**Figure 17.**
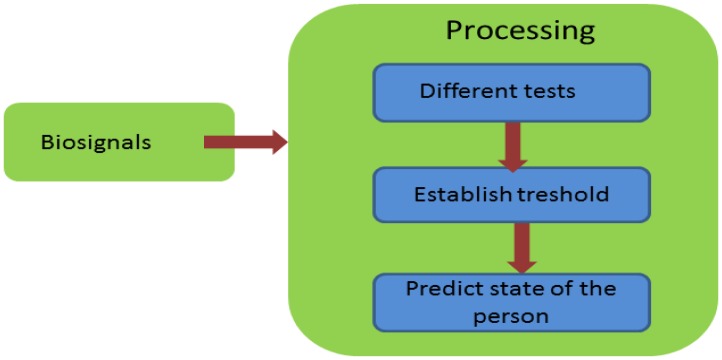
Block C—stress detection.

The structure of the stress application is represented in Figure 17.

## 5. Results

### 5.1. Monitoring

**Figure 18.**
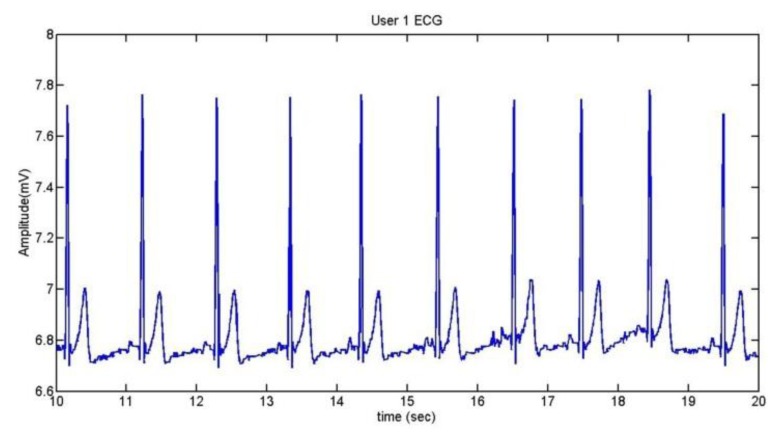
User 1 ECG.

**Figure 19.**
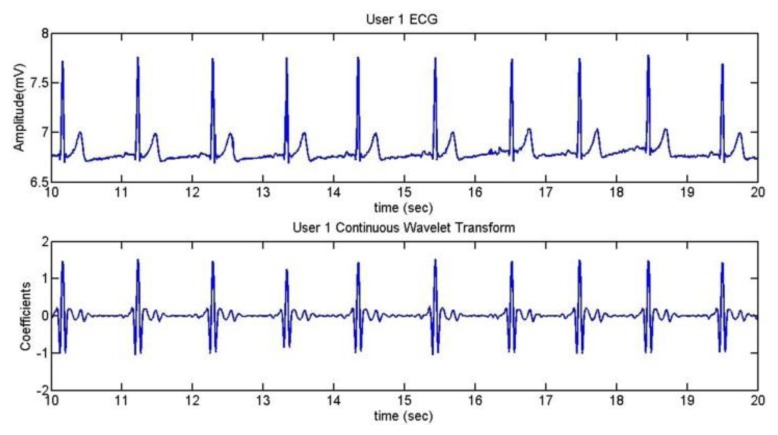
User 1 ECG and CWT.

To extract the information of the ECG, we take the R wave as a reference [41]. The method used is the Continuous Wavelet Transform (CWT). After doing some trials with different kinds of wavelets, it was established that the wavelet coif5 is the best one for the application. Figure 18 shows the ECG of User 1 during 10 seconds and the comparison between the ECG and its CWT is represented in Figure 19.

**Figure 20.**
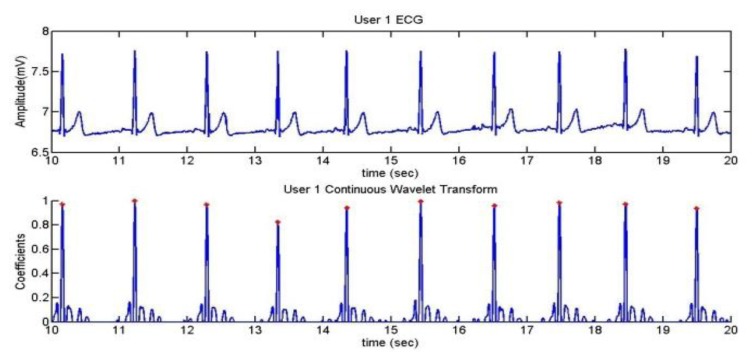
Detected peaks.

The pulsimeter shows clearly the QRS complex; so, after using the CWT algorithm, the R peaks can be well appreciated. In Figure 20, a mark has been added in order to find out whether the algorithm has detected the R wave correctly.

The confusion matrix of the detection of the R wave for each user is represented in Tables 2, 3, 4 and 5. The well detected R peaks are considered as True Positives, whereas the classification of a peak that should not be R is considered as False Negative.

By means of the confusion matrix, the sensitivity, the specificity and the precision were calculated in order to check the algorithm. The results for each user are shown in Tables 6 and 7. The average accuracy of the algorithm is 99.42%.

The sensitivity is 95.12% in the worst case but the specificity of user 1 is 100%, with a precision of 100%. For all the cases, the relation between the specificity and the sensitivity is very acceptable.

### 5.2. Detection of Anomalies

**Figure 21.**
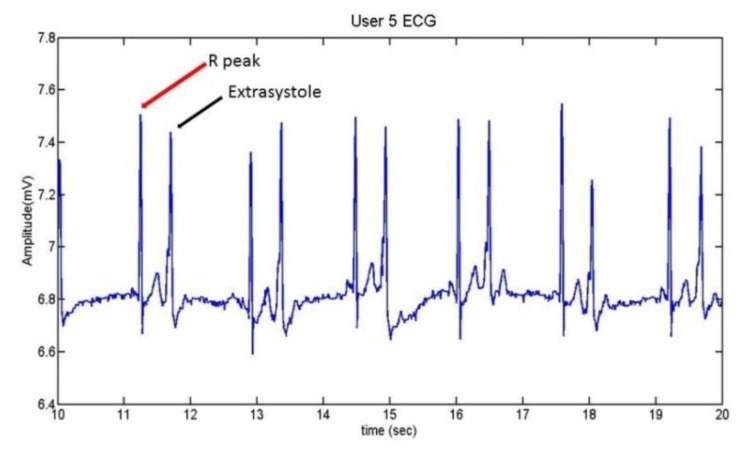
User 5 ECG with extrasystoles.

**Figure 22.**
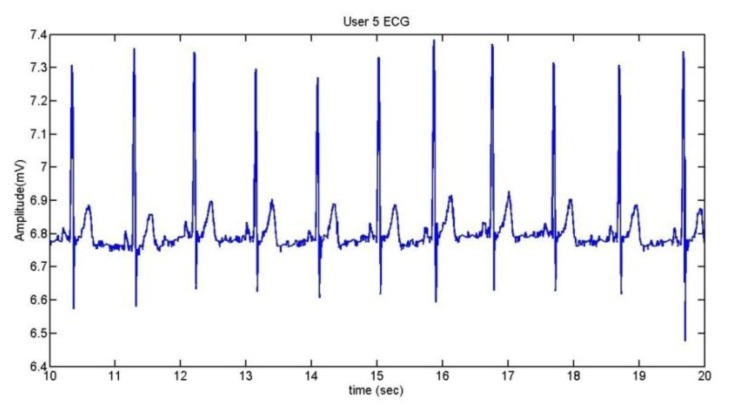
User 5 normal ECG.

User 5 is the person who suffers extrasystoles. The ECG which presents this anomaly can be seen in Figure 21, while Figure 22 contains her normal ECG.

A comparison between the two graphs indicates that the heart frequency is lower when the user presents extrasystoles. In this case, for the same period of time (10 seconds), the heart frequency presenting extrasystoles is the half of a normal ECG.

**Figure 23.**
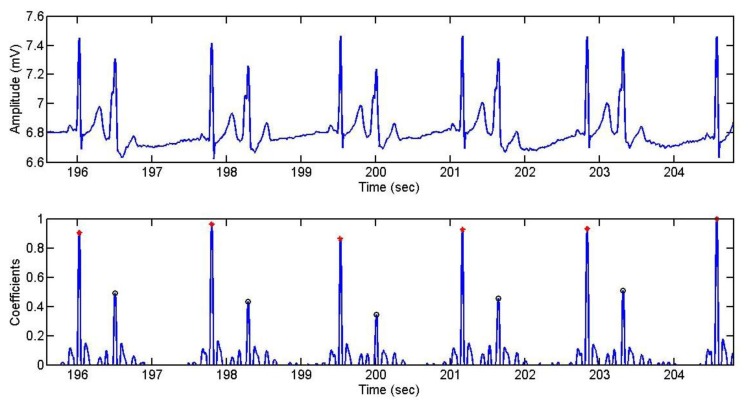
R wave and extrasystole detection.

Below, Figure 23 shows the detection of the R wave and the extrasystoles by the algorithm. Four different tests were recorded, corresponding to four different days. The red points represent the peaks of the R wave, while the black circles represent the peak of the extrasystole.

The confusion matrix for this case is shown in Table 8 and the average in Table 9.

**Figure 24.**
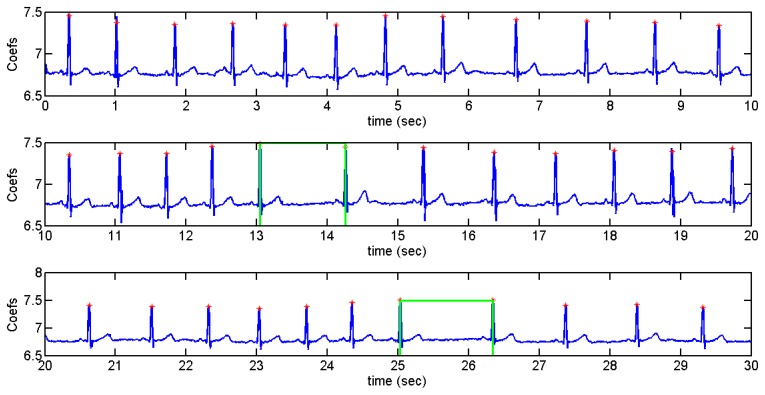
Arrhythmias detected in User 2.

**Figure 25.**
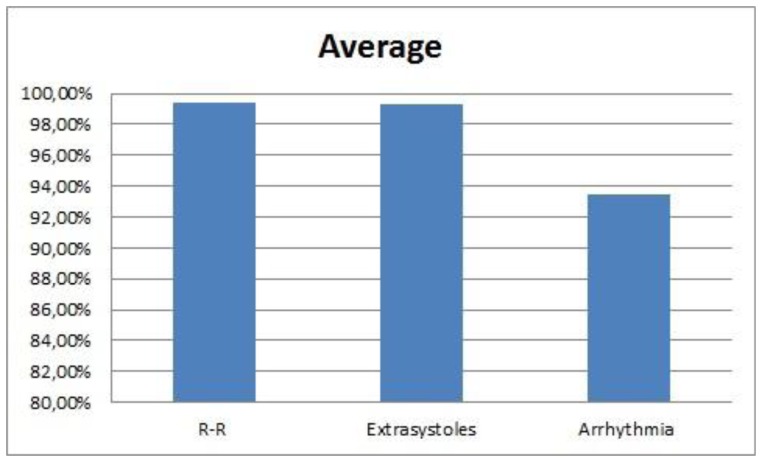
Success rate of the CWT.

User 2 had arrhythmias in her ECG. The recordings were done in three different days. Figure 24 shows the arrhythmias detected by a green rectangle. The graph shows that the time interval between pulses is longer when there is an arrhythmia. Confusion matrix and statistical results are in Tables 10 and 11, respectively:

The average of the CWT for the R–R interval and the anomalies is shown in Figure 25.

### 5.3. Stress Detection

The two trials explained before are used in order to induce a state similar to stress in the users, but some of the users have pointed that they have not felt a stressful sensation. After performing each trial, the results show that not all the users have been stressed or annoyed by the different tests. In order to point the classifier which signals should be considered as positive (stress) and which ones as negative (relax) the different users have been asked to indicate in which part they experienced any reaction.

In the case of the Stroop test, the criteria have been the following:

- The samples while the users are doing the test are considered as positive
- The negative samples have been chosen depending whether the user has been more relaxed at the beginning (first 55 seconds) or at the end (last 60 seconds).

The case of the user 12 was different due to the fact that he was not completely relaxed neither at the beginning nor at the end, so the range of the negative samples was between 15 and 55 seconds.

The criterion to separate between positives and negatives in the Sounds test has been done according to the users' opinion. It has not been possible to separate them objectively because some of the users have not presented any reaction. Moreover, other users have reacted only in different parts of the test.

#### 5.3.1. SCR

The confusion matrix of the SCR at the Stroop test can be seen in Tables 12 and 13. This test was done by five of the users and the results have been classified using J48 in Weka. The skin variability depends on each person, so the threshold is different for each user.

As said before, user 12 is a special case, so the corresponding number of samples is less than for the rest of the users. The classification averages can be seen in Table 14.

**Figure 26.**
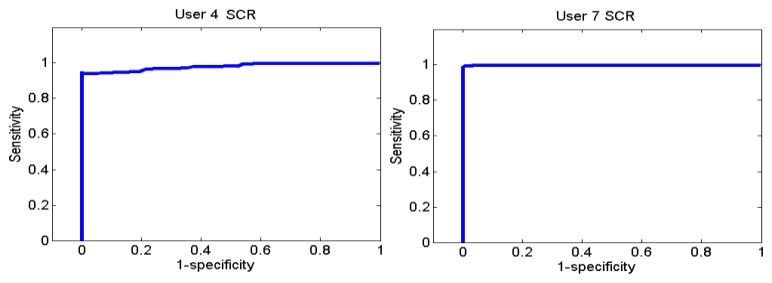
ROC curve of users 4 and 7 from the Stoop Test.

**Figure 27.**
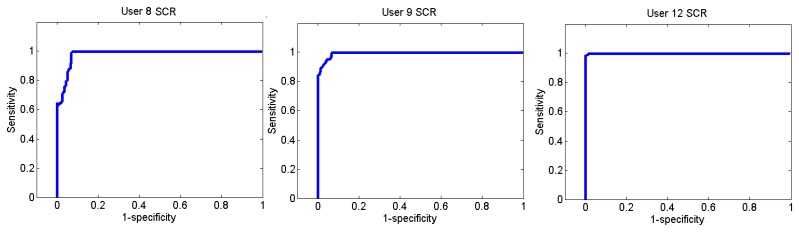
ROC curve of users 8, 9 and 12 from the Stoop Test.

Figures 26 and 27 show the ROC curves for each user. Each graph represents the False Positives against the True Positives. The ROC curve shows the values of the sensitivity and the specificity in order to verify if the classifier presents points with high sensitivity and high specificity. The best result is the one that presents a coordinate on the top and on the left, because it means that the specificity and the sensitivity are 100%.

User 8 is the one who has presented higher errors, so her graph shows that, to obtain 100% of sensitivity, the specificity decreases. However, the coordinate on the left and on the top shows that for a sensitivity of 100%, the specificity is 92.7%, so it is acceptable. In the case of user 9, to get a sensitivity of 100%, the specificity is 93.1%.

Users 4 and 8 indicated that they were more relaxed at the beginning and users 7 and 9 indicated they were more relaxed at the end. As it has been said before, user 12 is a special case.

**Figure 28.**
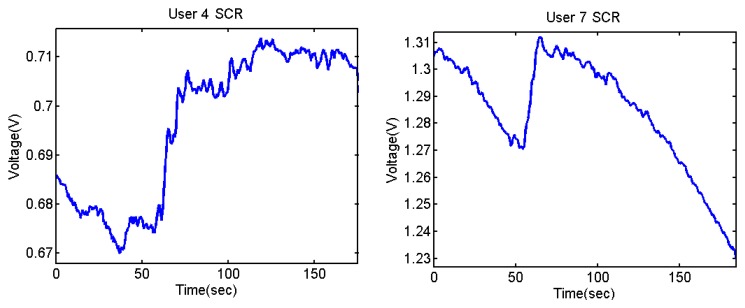
Skin response of users 4 and 7 from Stoop Test.

**Figure 29.**
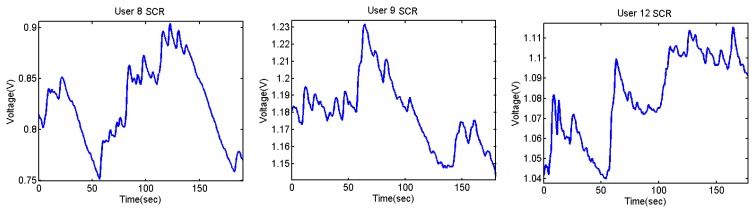
Skin response of users 8, 9 and 12 from Stoop Test.

The output voltages of the SCR for the Stroop test are shown in Figures 28 and 29. In them, there is an increment of the output voltage around second 60 due to the fact skin resistance decreases. User 7 is a special case because he did not encounter any difficulty in doing the Stroop test, so despite the fact that his skin resistance decreases at the beginning, then it starts to decrease until the end.

The difference between being stressed or relaxed differs only in terms of millivolts, but the resolution of the converter is good enough to detect these stages. It is noteworthy that after 55–60 seconds, there is an increment of the output signal.

Table 15 shows the average of the obtained values of the SCR, indicating the sex and the age of each user.

As regards listening to annoying sounds, not all the users reacted. This part was carried out by ten of the users. For three of them, the sounds were played at a low volume in order to know whether the skin reacts to all sounds or just to the annoying ones. These three users did not notice any significant difference when listening to different sounds. For the rest of them, the data were analysed with Weka and the J48 classifier. Tables 16^17^, 18 and 19 show the confusion matrix and the average of the classifier.

The samples for training the classifier were separated taking the users' opinions into account. Only those sounds that caused a reaction were classified as ‘stressful’. In Table 16, user 2 presents just 33 True Positive samples; this indicates that she has only reacted to one of the different sounds.

**Figure 30.**
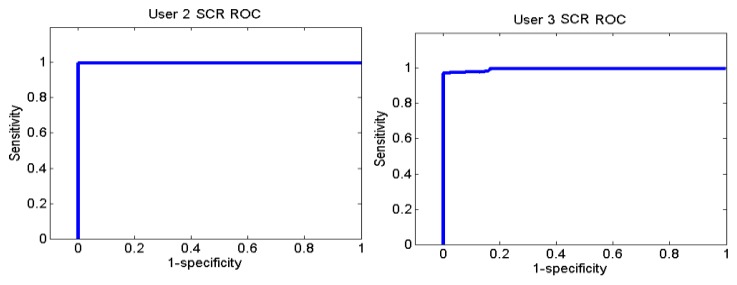
ROC curve of users 2 and 3 from Sounds Test.

**Figure 31.**
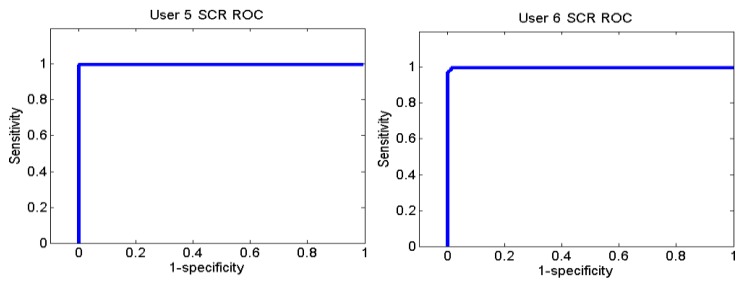
ROC curve of users 5 and 6 from Sounds Test.

**Figure 32.**
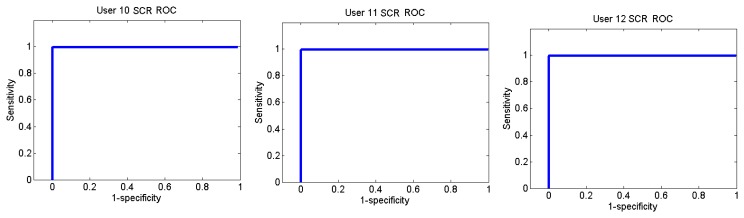
ROC curve of users 10, 11 and 12 from Sounds Test.

The ROC curves of the different users are shown in Figures 30, 31 and 32.

**Figure 33.**
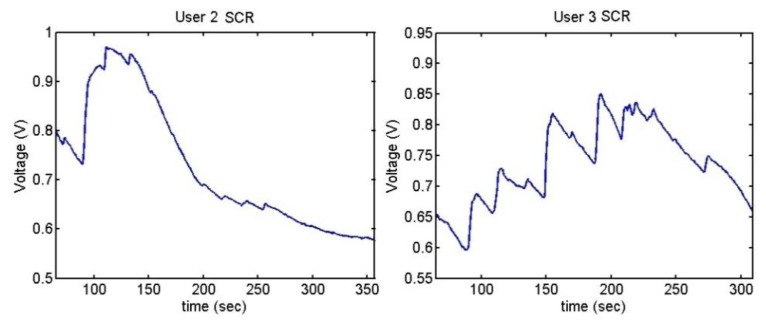
Skin response of users 2 and 3 from Sounds Test.

**Figure 34.**
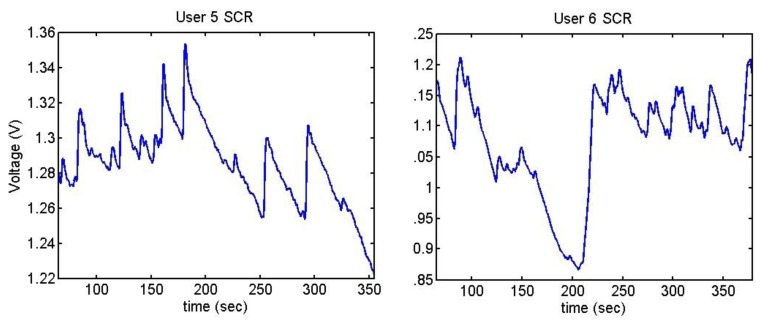
Skin response of users 5 and 6 from Sounds Test.

**Figure 35.**
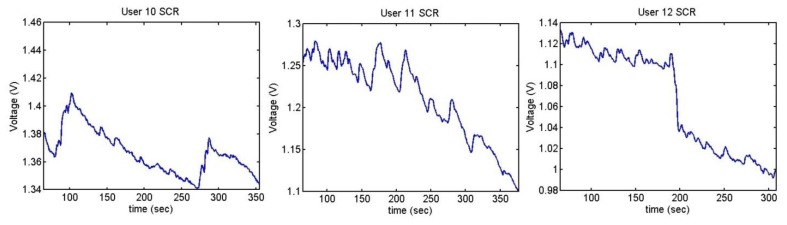
Skin response of users 10, 11 and 12 from Sounds Test.

Figures 33, 34 and 35 show the variation of the Skin Response of the different users at the time of listening annoying sounds:

The sudden increment of the signal indicates the stage where that has happened. User 2 has only reacted to one of the sounds and then, her skin resistance has increased because she has been relaxed. User 6 shows high reaction to one of the sounds, which has conditioned her until the end of the test.

**Figure 36.**
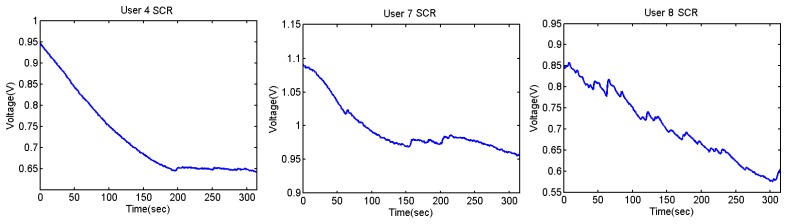
Skin response for users 4, 7 and 8. The volume of the sounds has been less than for the rest of the users.

It can be seen that the output voltage increases each time the users hear a sound and decreases after hearing it. The graphs of users 3 and 5 clearly show the peaks. As mentioned, three of the users have done the test with low volume, without presenting any change. Figure 36 shows their outputs.

The skin resistance has been increased during the test, which means that they have been more relaxed. Only user 8 presents little reactions to the sounds, but they have not been considered as valid because they are not really significant. The table below, Table 20, shows the mean of the output voltage for the different users, indicating the age and the sex.

Table 21 shows the average of the obtained results in the two trials and the total average of both of them.

#### 5.3.2. Pulsimeter

The ECG has not shown any significant differences between the state of stress and feeling relaxed in both tests, but only in one of them. The average and the deviation of the heart rate have been analysed in both cases.

Stroop test: there is no difference in the averages of the R–R intervals between the users doing the test and when they feel in calm. On the other hand, the users presented a decrease in the deviation while they were carrying out the Stroop test, Table 22.

**Figure 37.**
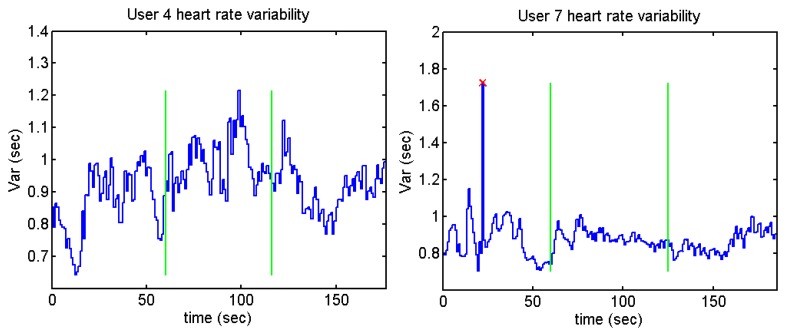
Heart rate variability of User 4 from Stroop Test.

**Figure 38.**
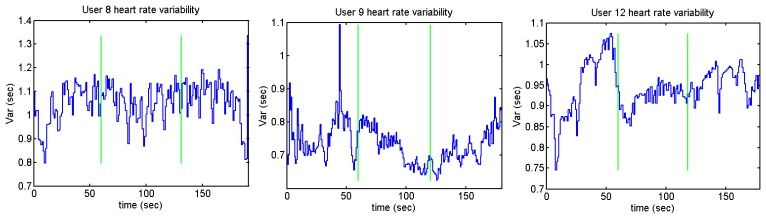
Heart rate variability of User 12 from Stroop Test.

Despite the fact that the standard deviation is higher before and after doing the test (except for user 12), only one of the stages has been taken into account as a relaxed stage: before or after doing the test (depending on the users' opinion). Figures 37 and 38 show the heart rate variability of the different users while they were performing the Stroop test. The time between each R peak is represented by the y-axis, while x-axis represents the whole time of the test.

**Figure 39.**
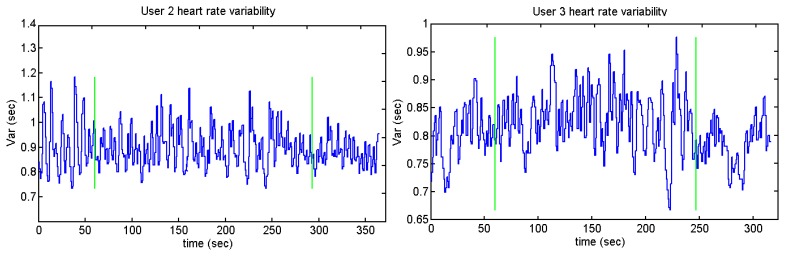
Heart rate variability of users 2 and 3 from Sounds Test.

**Figure 40.**
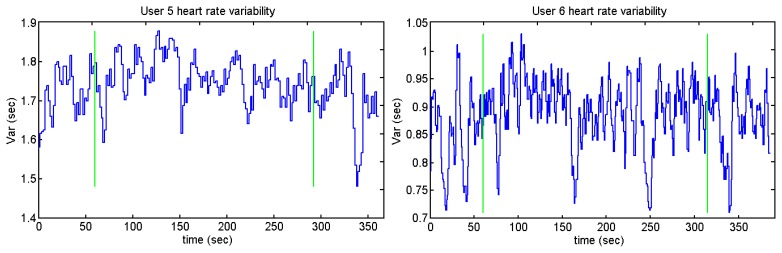
Heart rate variability of users 5 and 6 from Sounds Test.

**Figure 41.**
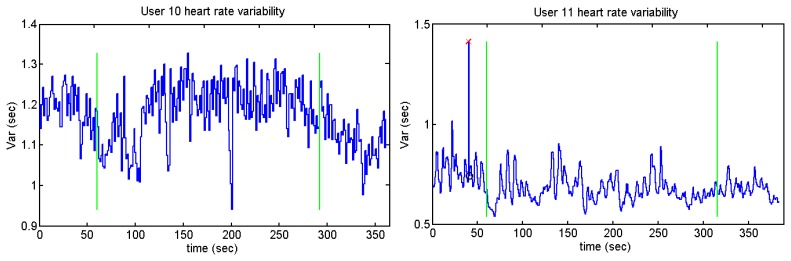
Heart rate variability of users 10 and 11 from Sounds Test.

**Figure 42.**
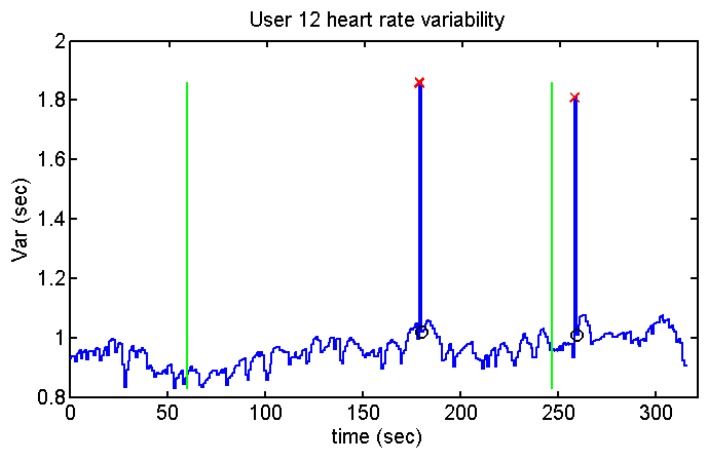
Heart rate variability of User 12 from Sounds Test.

Users 7 and 9 present a high peak in the heart variability: this means that there is an R-R peak which has not been detected. The annoying sounds test does not show any difference concerning the average and standard deviation. This could be because the length of the annoying sounds was short and the interval between the different sounds is not long enough. Figures 39, 40, 41 and 42 show the heart rate variability in the Sound test:

Those users who present higher heart rates contain more samples than the ones whose pulse number is lower. It can be appreciated by users 2 and 10 against user 5, who is the person with the lesser number of pulses per minute. The graph of user 11 has the same peak as users 7 and 9. Figures 41 and 42 display a cross and a circle as the algorithm indicates that the previous algorithm has not detected an R peak.

## 6. Discussion

The ECG used has two principal functions: detection of anomalies and detection of stress wearing one only device. For the second function, a SCR was used to verify the results. The chosen device is a BioHarness pulsimeter, designed by Zephyr. The ECG signals have been obtained from 12 healthy workers of Deusto University. The sample group is integrated by five women and seven men aged between 23 and 35. In the case of the remote monitoring and detection of anomalies, the same algorithm with the same characteristics has been used. For the third point, stress level, the J48 classifier has been used, but different thresholds have been applied for each one of the users due to the fact that the skin resistance presents different variability depending on the person. Hence, it does not matter the physical differences between one user and other: each classifier is designed for each user.

The results obtained have a similar success than studies that used MIT-BIH Arrhythmia Database: 96.65% in [42], 98.81% for T wave and ST segment detection in [43] and 99.64% in [44].

The analysis of the ECG shows the heart rate (detecting the R wave) and possible anomalies in addition to a decrement of the standard deviation of the heart rate variability in the Stroop test when the user is doing the test. On the contrary, it does not indicate any significant change when the user is relaxed or stressed in the Sounds test. For the analysis of the ECG, the Continuous Wavelet Transform (CWT) was used. This method is not influenced by the motion of the user so the analysis can be done when the user is practicing sport. In [25] the authors use CWT with a success of the 99.5%.

The algorithm detects the R peaks with a success higher than the 99% and with a sensitivity of 95.12% in the worst case. There are also users who have presented a sensitivity of 100%. The specificity is the 99.9%, so there is a low number of False Positives.

This wave is then taken as reference to obtain the heart rate and the possible anomalies. The detection of the R wave is done using a frequency method, thus not being necessary to obtain training samples before, although the results applying machine learning are also over the 99% [23].

The detection of anomalies has also produced good results, with a sensitivity of 99.29% in the case of extrasystoles and 93.48% in arrhythmias. For both cases, the specificity is 100%. The same algorithm has been used for all the users and no extrasystole or arrhythmia has been detected in the rest of the users: only in 5 and 2, respectively.

After the detection of the R wave and after verifying the correct classification, the next stage has been to find out whether the pulsimeter could detect stress emotion. The method is based on the heart rate variability, so the time between each peak is analysed.

The results obtained by the Stroop test indicate that the standard deviation is less while the users are doing the test, but the Sounds test does not show any difference. The results obtained by the SCR are only used to verify whether the users have presented any reaction to the different tests and to find out in which stages they have presented significant differences.

In order to establish stress levels we have taken into consideration the personal opinion of the users because each one felt stressed in different parts of the trials: it has not been possible to establish a threshold with objective trials. However, the intention of this study is to verify whether it is possible to detect stress using only a commercial pulsimeter not to develop a system which is able to detect whether a person is stressed. Nevertheless, the two tests were developed as applications which could be executed at users' home to obtain objective results.

The Skin Conductance Response produced results with a high average of success in those subjects that experienced any reaction to different tests. In all cases contained in the present study, the classifier establishes thresholds which are over 80%, 94.02% being the total average. However, the heart rate did not undergo any significant changes during the Sounds test. A diminution of the standard deviation was appreciated in the Stroop test.

Since one of the final objectives is to develop an application which is able to establish the different stress levels of the users at home with the pulsimeter, the trials were short in terms of duration. This could be the reason why there are no differences between the averages of the heart rate at the stages where the user should be stressed listening to annoying sounds.

## 7. Conclusions

The algorithm developed detects the heart rate with and sensitivity of 99.42%, a sensitivity of 95.12% in the worst case and a specificity of 99.9%. Extrasystoles have been detected with a sensitivity of 99.29% and a specificity of 100%. In the case of the arrhythmias, the sensitivity has been 93.48%.

The base of the developed algorithm is the CWT, so this application enables the detection of ECG oddities in real time without being influenced the user's movements. The device used for the study of the ECG is a commercial device which can be worn by the users while they are playing sports. It is indeed very comfortable for those cases where it is necessary to study the reaction of the body in situations requiring effort. As the device communicates via Bluetooth, it would be possible to implement this algorithm in a mobile phone.

Hence, the two first points of the study (remote monitoring and detection of anomalies) can be implemented with the Zephyr pulsimeter.

Stress detection produced different results depending on the users because some of them have not experienced any reaction to the different trials. As far as the Stroop test is concerned, the standard deviation of the heart rate variability is lower when the users are doing the test than when they are not. The Sounds test does not show any significant difference.

Comparing the SCR with the Heart Rate, it can be said that, with these trials, the skin presents wider differences between the stages where the user is supposed to be relaxed and when they are supposed to be stressed. Some stress stages which have been detected by the GSR have not been detected by the pulsimeter.

Concerning the heart rate, the reason why there is not a significant difference could be because the different trials used to stress the users were short. The Stroop test has submitted the users to longer stress periods than the Sounds test. Furthermore Stroop test has presented differences with respect to the heart rate: the standard deviation in normal stages is higher than in stress stages. This could mean that the heart needs more time to react than the skin resistance but, in long periods, the difference of the heart rate is appreciable.

The next action lines of this study are:

- Detection of more anomalies: this method allows analysis of the QRS complex in order to detect any other anomalies that may appear. By taking the R wave as a reference and studying different types of heart diseases, it would be possible to increase the number of anomalies the application is able to detect.
- A longer test in order to distinguish the emotional state of the user: the two trials did not produce the expected results, which could be because there is not enough time between calm situations and stressing factors. The authors think that if longer intervals of calm were established, the results could be different.

## Figures and Tables

**Table 1.** User descriptions.

	**Age**	**Sex**		**Age**	**Sex**
**User 1**	25	Male	**User 7**	25	Male
**User 2**	26	Female	**User 8**	23	Male
**User 3**	24	Female	**User 9**	23	Female
**User 4**	26	Male	**User 10**	24	Male
**User 5**	27	Female	**User 11**	30	Male
**User 6**	25	Female	**User 12**	35	Male

**Table 2.** Confusion matrix for the R wave I.

**User 1**			**User 2**			**User 3**		
	P	N		P	N		P	N
T	117	6	T	538		T	519	0
F	0	32,901	F	0	101,606	F	0	101,625

**Table 3.** Confusion matrix for the R wave II.

**User 4**			**User 5**			**User 6**		
	P	N		P	N		P	N
T	217	1	T	229	2	T	444	0
F	0	53,458	F	10	101,903	F	3	101,697

**Table 4.** Confusion matrix for the R wave III.

**User 7**			**User 8**			**User 9**		
	P	N		P	N		P	N
T	523	0	T	292	0	T	245	0
F	0	101,621	F	0	78,710	F	0	44,800

**Table 5.** Confusion matrix for the R wave IV.

**User 10**			**User 11**			**User 12**		
	P	N		P	N		P	N
T	306	0	T	551	1	T	327	2
F	0	90,162	F	0	95,460	F	0	78,610

**Table 6.** Statistics results I.

	**User 1**	**User 2**	**User 3**	**User 4**	**User 5**	**User 6**
**Sensitivity**	0.9512	1	1	0.9954	0.9913	1
**Specificity**	1	1	1	1	0.9999	0.99997
**Precision**	0.9998	1	1	0.99998	0.9999	0.99997

**Table 7.** Statistics results II.

	**User 7**	**User 8**	**User 9**	**User 10**	**User 11**	**User 12**
**Sensitivity**	1	1	1	1	0.9982	0.9939
**Specificity**	1	1	1	1	1	1
**Precision**	1	1	1	1	0.99999	0.99997

**Table 8.** Confusion matrix for esxtrasystoles detection for user 5.

Test 1			Test 2			Test 3			Test 4		
	P	N		P	N		P	N		P	N
T	189	0	T	348	0	T	225	8	T	362	0
F	0	210	F	0	702	F	0	236	F	0	722

**Table 9.** Statistic results for extrasystoles detection.

	**Sensitivity**	**Specificity**
Test 1	100%	100%
Test 2	100%	100%
Test 3	96.57%	100%
Test 4	100%	100%
**Total**	99.29%	100%

**Table 10.** Confusion matrix for arrhythmia detection of user 2.

Test 1			Test 2			Test 3		
	P	N		P	N		P	N
T	16	1	T	7	0	T	20	2
F	0	517	F	0	401	F	0	318

**Table 11.** Statistics results for arrhythmia detection.

	**Sensitivity**	**Specificity**
Test 1	94.12%	100%
Test 2	100%	100%
Test 3	90.91%	100%
**Total**	93.48%	100%

**Table 12.** SCR confusion matrix for Stroop test I.

**User 4**			**User 7**			**User 8**		
	P	N		P	N		P	N
T	221	15	T	234	2	T	134	4
F	1	212	F	1	231	F	18	215

**Table 13.** SCR confusion matrix for Stroop test II.

**User 9**			**User 12**		
	P	N		P	N
T	165	1	T	235	1
F	16	216	F	3	155

**Table 14.** SCR average from Stroop test.

	**User 4**	**User 7**	**User 8**	**User 9**	**User 12**
**Correct**	96.44%	99.34%	94.07%	95.72%	98.98%
**Incorrect**	3.56%	0.641%	5.93%	4.27%	1.02%
**Error**	12.91%	2.08%	18.54%	15.61%	3.14%
**Success**	87.09%	97.92%	81.46%	84.39%	96.86%

**Table 15.** Mean values of the SCR from Stroop test.

	**Age**	**Sex**	**Stress (V)**	**Relax (V)**
**User 4**	26	Male	0.7026	0.6775
**User 7**	25	Male	1.3011	1.2647
**User 8**	23	Male	0.8597	0.8093
**User 9**	23	Female	1.2106	1.1834
**User 12**	35	Male	1.0849	1.055

**Table 16.** SCR confusion matrix for annoying sounds I.

**User 2**			**User 3**			**User 5**			**User 6**		
	P	N		P	N		P	N		P	N
T	33	0	T	425	7	T	707	0	T	228	3
F	1	683	F	4	294	F	1	319	F	6	391

**Table 17.** SCR confusion matrix for annoying sounds II.

**User 10**			**User 11**			**User 12**		
	P	N		P	N		P	N
T	437	0	T	715	0	T	511	0
F	1	507	F	3	353	F	1	450

**Table 18.** Average of annoying sounds I.

	**User 2**	**User 3**	**User 5**	**User 6**
**Correct**	99.86%	98.49%	99.90%	98.57%
**Incorrect**	0.14%	1.51%	0.10%	1.43%
**Error**	1.57%	4.31%	0.23%	4.60%
**Success**	98.43%	95.69%	99.77%	95.40%

**Table 19.** Average of annoying sounds II.

	**User 10**	**User 11**	**User 12**
**Correct**	99.89%	99.72%	99.90%
**Incorrect**	0.11%	0.28%	0.10%
**Error**	0.21%	0.63%	0.21%
**Success**	99.79%	99.37%	99.79%

**Table 20.** Mean values of the SCR from annoying sounds.

	**Age**	**Sex**	**Stress (V)**	**Relax (V)**
**User 2**	26	Female	1.1370	0.6312
**User 3**	24	Female	0.794	0.6549
**User 5**	27	Female	1.2971	1.232
**User 6**	25	Female	1.137	0.9741
**User 10**	24	Male	1.3824	1.3482
**User 11**	30	Male	1.2483	1.1457
**User 12**	35	Male	1.1113	1.0158

**Table 21.** Average by Test and total.

	**Stroop Test**	**Anoying sounds**	**Total**
**Average**	89.74%	98.32%	94.02%

**Table 22.** Standard deviation of the heart rate in Stroop test.

	**User 4**	**User 7**	**User 8**	**User 9**	**User 12**
**Before test**	0.0697	0.0831	0.1473	0.0429	0.1015
**During test**	0.0527	0.0268	0.0413	0.0310	0.0789
**After test**	0.0502	0.0308	0.0585	0.0551	0.0720

## References

[1] Gumbinger C., Krumsdorf U., Veltkamp R., Hacke W., Ringleb P. (2012). Continuous monitoring versus HOLTER ECG for detection of atrial fibrillation in patients with stroke. Eur. J. Neurol..

[2] Jocham H.R., Dassen T., Widdershoven G., Middel B., Halfens R. (2009). The Effect of Palliative Care in Home Care and Hospital on Quality of Life. Hosp. Palliat. Nurs. J..

[3] Fukui S., Fujita J., Tsujimura M., Sumikawa Y., Hayashi Y. (2011). Predictors of home death of home palliative cancer care patients: A cross-sectional nationwide survey. Int. J. Nurs. Stud..

[4] Lee R.G., Chen K.C., Hsiao C.C., Tseng C.L. (2007). A mobile care system with alert mechanism. IEEE Trans. Inf. Technol. Biomed..

[5] Kurl S., Mäkikallio T.H., Rautaharju P., Kiviniemi V., Laukkanen J.A. (2012). Duration of QRS complex in resting electrocardiogram is a predictor of sudden cardiac death in men. Circ. J..

[6] Steptoe A., Ayers S. (2005). Stress, Health and Illness. The SAGE Handbook of Health Psychology.

[7] Zhao Z., Cui L. EasiMed: A Remote Health Care Solution.

[8] Jung J.Y., Lee J.W. ZigBee Device Access Control and Reliable Data Transmission in ZigBee Based Health Monitoring System.

[9] Führ H. Abstract Harmonic Analysis of Continuous Wavelet Transforms.

[10] Armario P., Hernández del Rey R., Martín-Baranera M. (2002). Estrés, enfermedad cardiovascular e hipertensión arterial. Med. Clin..

[11] Karthikeyan P., Murugappan M., Yaacob S. A Review on Stress Inducement Stimuli for Assessing Human Stress Using Physiological Signals.

[12] Salahuddin L., Cho J., Jeong M.G., Kim D. Ultra Short Term Analysis of Heart Rate Variability for Monitoring Mental Stress in Mobile Settings.

[13] Varshney U. Pervasive Healthcare Computing: EMR/HER, Wireless and Health Monitoring.

[14] Saponara S., Donati M., Fanucci L., Sanchez-tato I., Carmona C. Remote Monitoring of Vital Signs in Patients with Chronic Heart Failure Sensor Devices and Data Analysis Perspective.

[15] Bifulco P., Gargiulo G., Romano M., Fratini A., Cesarelli M. Bluetooth Portable Device for Continuous ECG and Patient Motion Monitoring During Daily Life.

[16] Gil Y., Wu W., Lee J. (2012). A Synchronous Multi-Body Sensor Platform in a Wireless Body Sensor Network: Design and Implementation. Sens. J..

[17] Lee Y.-D., Chung W.-Y. (2009). Wireless sensor network based wearable smart shirt for ubiquitous health and activity monitoring. Sens. Actuat. B Chem..

[18] Mura G. (2008). Wearable technologies for emotion communication. Middle East Tech. Univ. J. Archit..

[19] Pandian P.S., Mohanavelu K., Safeer K.P., Kotresh T.M., Shakunthala D.T, Gopal P., Padaki V. (2008). C. Smart vest: Wearable multi-parameter remote physiological monitoring system. Med. Eng. Phys..

[20] Pandey B., Mishra R.B. (2010). An integrated intelligent computing method for the detection and interpretation of ECG based cardiac diseases. Int. J. Knowl. Eng. Soft Data Paradig..

[21] Köhler B.U., Hennig C., Orglmeister R. (2002). The principles of software QRS detection. IEEE Eng. Med. Boil. Mag..

[22] Benitez D., Gaydecki P.A., Zaidi A. (2001). ; Fitzpatrick A.P. The use of the Hilbert transform in ECG signal analysis. Comput. Biol. Med..

[23] Mehta S.S., Lingayat N.S. (2007). Support vector machine for cardiac beat detection in single lead electrocardiogram. IAENG Int. J. Appl. Math..

[24] Janusek D., Kania M., Zaczek R., Opolski G., Maniewski R. (2011). Application of wavelet based denoising for T-wave alternans analysis in high resolution ECG maps. Meas. Sci. Rev. J..

[25] Alvarado C., Arregui J., Ramos J., Pallàs-areny R. Automatic Detection of ECG Ventricular Activity Waves using Continuous Spline Wavelet Transform.

[26] Gautam A., Kaur M. (2012). ECG analysis using continuous wavelet transform. IOSR J. Eng..

[27] Abibullaev B., Kang W., Lee S., An J. (2010). A simple wavelet method for automated detection of epileptic neural spikes in electroencephalogram. Int. J. Adv. Inf. Sci. Serv. Sci..

[28] Minchev H., Dukov G., Georgiev S. (2009). EEG spectral analysis in serious gaming: An *ad hoc* experimental application. Int. J. Bioatumation.

[29] Jan Y.K., Struck B.D., Foreman R.D., Robinson C. (2009). Wavelet analysis of sacral skin blood flow oscillations to assess soft tissue viability in older adults. Microvasc. Res..

[30] Brawner K.W., Goldberg B.S. Real-Time Monitoring of ECG and GSR Signals during Computer-Based Training.

[31] Healey J.A., Picard R.W. (2005). Detecting stress during real-world driving tasks using physiological sensors. IEEE Trans. Intell. Transp. Syst..

[32] Carter J.R., Ray C.A. (2009). Sympathetic neural responses to mental stress: Responders, nonresponders and sex differences. Am. J. Physil. Heart Circ. Physiol..

[33] Jovanov E., O'Donnell Lords A., Raskovic D., Cox P.G., Adhami R., Andrasik F. (2003). Stress monitoring using a distributed wireless intelligent sensor system. IEEE Eng. Med. Biology Mag..

[34] Jongyoon C., Gutierrez-Osuna R. (2011). Removal of Respiratory Influences from Heart Rate Variability in Stress Monitoring. Sens. J..

[35] Santos A., Sánchez C., Guerra J., Bailador Del Pozo G. (2011). A Stress-Detection System Based on Physiological Signals and Fuzzy Logic. IEEE Trans. Industr. Electrs..

[36] Shi Y., Nguyen M.H., Blitz P., French B., Fisk S., De la Torre F., Smailagic A., Siewiorek D. Personalized Stress Detection from Physiological Measurements.

[37] Liao W., Zhang W., Zhu Z., Ji Q. A Real-Time Human Stress Monitoring System Using Dynamic Bayesian Network. In Proceedings of IEEE Computer Society.

[38] Bardzell S., Bardzell J., Pace T. Understanding Affective Interaction: Emotion, Engagement, and Internet Videos.

[39] Villarejo M.V., Zapirain B.G., Zorrilla A.M. (2012). A stress sensor based on Galvanic Skin Response (GSR) controlled by ZigBee. Sens. J..

[40] Duque E., Rivera J.H., Vera O.E. (2006). Extracción de características de la señal electrocardiográfica mediante software de análisis matemático. Sci. Tech..

[41] Haque A., Hnif M.D. (2009). ; Adnan M. Detection of small variations of ECG features using Wavelet. ARPN J. Eng. Appl. Sci..

[42] Pachauri A., Bhuyan M. (2009). Robust detection of r-wave using wavelet technique. World Acad. Sci. Eng. Technol..

[43] Gutiérrez A., Lara M., Hernández P.R. (2005). Evaluación de un Detector de Complejo QRS Basado en la Wavelet de Haar, Usando las Bases de Datos MIT-BIH de Arritmias y Europea del Segmento ST y de la Onda T. Comput. Sist..

[44] Benitez D.S., Gaydecki P.A., Zaidi A., Fitzpatrick A.P. A new QRS detection algorithm based on the Hilbert transforms.

